# Constructing functional cuticles: analysis of relationships between cuticle lipid composition, ultrastructure and water barrier function in developing adult maize leaves

**DOI:** 10.1093/aob/mcz143

**Published:** 2019-10-21

**Authors:** Richard Bourgault, Susanne Matschi, Miguel Vasquez, Pengfei Qiao, Annika Sonntag, Caleb Charlebois, Marc Mohammadi, Michael J Scanlon, Laurie G Smith, Isabel Molina

**Affiliations:** 1 Department of Biology, Algoma University, Sault Ste. Marie, ON, Canada; 2 Section of Cell and Developmental Biology, University of California San Diego, La Jolla, CA, USA; 3 Plant Biology Section, School of Integrative Plant Science, Cornell University, Ithaca, NY, USA

**Keywords:** Cuticular wax, cuticle ontogeny, cutin, cuticle ultrastructure, leaf development, maize, *Zea mays *

## Abstract

**Background and Aims:**

Prior work has examined cuticle function, composition and ultrastructure in many plant species, but much remains to be learned about how these features are related. This study aims to elucidate relationships between these features via analysis of cuticle development in adult maize (*Zea mays* L.) leaves, while also providing the most comprehensive investigation to date of the composition and ultrastructure of adult leaf cuticles in this important crop plant.

**Methods:**

We examined water permeability, wax and cutin composition via gas chromatography, and ultrastructure via transmission electron microscopy, along the developmental gradient of partially expanded adult maize leaves, and analysed the relationships between these features.

**Key Results:**

The water barrier property of the adult maize leaf cuticle is acquired at the cessation of cell expansion. Wax types and chain lengths accumulate asynchronously over the course of development, while overall wax load does not vary. Cutin begins to accumulate prior to establishment of the water barrier and continues thereafter. Ultrastructurally, pavement cell cuticles consist of an epicuticular layer, and a thin cuticle proper that acquires an inner, osmiophilic layer during development.

**Conclusions:**

Cuticular waxes of the adult maize leaf are dominated by alkanes and alkyl esters. Unexpectedly, these are localized mainly in the epicuticular layer. Establishment of the water barrier during development coincides with a switch from alkanes to esters as the major wax type, and the emergence of an osmiophilic (likely cutin-rich) layer of the cuticle proper. Thus, alkyl esters and the deposition of the cutin polyester are implicated as key components of the water barrier property of adult maize leaf cuticles.

## INTRODUCTION

Plant epidermal cells of the shoot are covered by a hydrophobic layer, the cuticle (Esau, 1977), which forms the primary barrier between the plant’s air-exposed surfaces and the external environment. The cuticle limits non-stomatal water loss and gaseous exchanges, protects the plant from extreme temperatures, UV radiation and pathogens, provides mechanical strength, and prevents organ fusion during development (Kolattukudy, 2001; Nawrath, 2002). These functions of the cuticle allowed plants to survive in terrestrial habitats early in land plant evolution, with fossil evidence showing cuticles in the earliest known land plants (Bargel *et al.*, 2006).

Plant cuticles contain a lipid biopolymer, cutin, which is embedded in intracuticular waxes and covered by epicuticular waxes (Koch and Ensikat, 2008). Cutin is a polyester matrix composed mainly of glycerol and long-chain (C_16_ and C_18_) fatty acid monomers, usually having ω-linked functional groups and often containing mid-chain hydroxy and epoxy groups. However, the native structure of cutin remains largely hypothetical due to the inability to extract and analyse cutin without prior depolymerization (Kolattukudy, 1980; Graça *et al.*, 2002; Heredia, 2003; Pollard *et al.*, 2008). Cuticular waxes are composed of a mixture of aliphatic and alicyclic compounds with diverse chemistries that are extractable with organic solvents (Samuels *et al.*, 2008; von Wettstein-Knowles, 2012). The aliphatic wax components are usually very-long-chain fatty acids, alkanes, alcohols, aldehydes, ketones and wax esters. Alicyclic components, including pentacyclic triterpenoids, tocopherols and steroids, are also frequently present (Jetter and Riederer, 2016). The specific composition and quantities of cuticular wax classes may vary significantly across species, plant organs and developmental stages, and may undergo dynamic effects in response to growth conditions, physical disturbance or damage, and genetic manipulation (Jenks *et al.*, 2001; Jetter *et al.*, 2006; Kosma *et al.*, 2009).

Ultrastructural studies have led to the definition of three principal layers of mature plant cuticles that are observable by transmission electron microscopy (TEM) (Jeffree, 1996; Yeats and Rose, 2013). The basal, cutin-rich ‘cuticular layer’ is recognized ultrastructurally by the presence of fibrils oriented perpendicularly to the plane of the cuticle that stain darkly with osmium tetroxide and consist of polysaccharides (Jeffree, 1996; Guzmán *et al.*, 2014; Mazurek *et al.*, 2017). External to the cuticular layer lies the ‘cuticle proper’ containing cutin impregnated with waxes, but considered to be devoid of carbohydrates and lacking the darkly staining fibrils that characterize the cuticular layer. However, recent studies highlighted the presence of cellulose and pectins in both layers (Guzmán *et al.*, 2014). Epicuticular wax constitutes the outermost layer of the cuticle. This layer may exist as a thin film, imparting a shiny or ‘glossy’ appearance, or may include crystals that impart a dull or ‘glaucous’ appearance if sufficiently abundant (Baker, 1982; Koch and Ensikat, 2008). Epicuticular wax is loosely associated with the rest of the cuticle and can be stripped off with adhesives such as gum arabic (Jetter and Schaffer, 2001), cellulose acetate or collodion (Zeisler and Schreiber, 2016), or by freezing in glycerol (Ensikat *et al.*, 2000). Analyses of epicuticular wax have shown that its composition is distinct from that of waxes embedded in the cuticle proper (‘intracuticular waxes’) (Jetter and Riederer, 2016; Zeisler-Diehl *et al.*, 2018). Understanding how these features of cuticle organization are related to its composition and function is an active area of investigation.

Impermeability to water is a critical feature of the plant cuticle. It is well established that waxes, rather than cutin, confer the majority of the water barrier property of the cuticle (Schönherr, 1976; Kerstiens, 2006; Isaacson *et al.*, 2009; Jetter and Riederer, 2016). Comparisons between closely related species and between mutant or transgenic versus wild-type individuals have shown that cuticle thickness has little impact on its function as a water barrier but that wax composition is critical; in particular, alicyclic waxes, including triterpenoids, tocopherols and steroids, increase the permeability of cuticles to water (Vogg *et al.*, 2004; Buschhaus and Jetter, 2012; Jetter and Riederer, 2016). Very few studies have analysed cuticle development as a way to investigate how compositional and structural features of the cuticle are related to its water barrier properties (Richardson *et al.*, 2007; Leide *et al.*, 2007).

Prior studies investigating the cuticles of maize leaves have mostly focused on juvenile leaves: the first six to eight leaves produced by the plant, depending on genotype (Foerster *et al.*, 2015). At maturity, juvenile leaves have a dense coating of epicuticular wax crystals that is absent from mature adult leaves produced later in the plant’s life (Sylvester *et al.*, 1990; Bongard-Pierce *et al.*, 1996). Waxes of mature, juvenile maize leaves are dominated by very-long-chain alcohols and aldehydes, with lower proportions of alkanes and esters (Bianchi *et al.*, 1978; Ristic and Jenks, 2002; Sturaro *et al.*, 2005; Javelle *et al.*, 2010; Loneman *et al.*, 2017). However, for agronomically important traits related to cuticle function, the adult leaf cuticle is likely of far greater significance. For example, drought stress is most damaging to grain yield during flowering and early kernel development (Grant *et al.*, 1989), by which time juvenile leaves have already senesced and only adult leaves remain. The cuticle composition of the adult maize leaf has not been extensively studied, although existing reports indicate that its wax composition differs substantially from that of juvenile leaves, with much higher proportions of wax esters and alkanes and smaller proportions of free alcohols and aldehydes (Bianchi *et al.*, 1984; Avato *et al.*, 1990; Yang *et al.*, 1993). In adult maize leaves, cutin is mainly composed of dihydroxyhexadecanoic acid and typical members of the C_18 _family of cutin acids, including hydroxy and hydroxy-epoxy acids (Espelie and Kolattukudy, 1979).

In this study, we exploit the developmental gradient of partially expanded maize leaves to investigate cuticle ontogeny in the adult maize leaf. We map the acquisition of water barrier properties onto this gradient and relate this functional maturation to changes in chemical composition and ultrastructure, yielding new insights into composition/structure/function relationships.

## MATERIALS AND METHODS

### Plant material and growth conditions

For cuticular lipid analysis, B73 maize (*Zea mays* L.) plants were grown in a 25 °C day and 20 °C night, in 60 % relative humidity with a 16 h:8 h light:dark photoperiod in controlled growth chambers until harvest at ~4 weeks. For functional maturation and TEM analysis, plants were grown in a glasshouse on the UCSD campus in La Jolla, CA (latitude 32.8856, longitude −117.2297), without supplementary lighting and with temperatures in the range of 18–30 °C. In preliminary experiments, wax and cutin profiles were found to be very similar at all developmental stages for plants grown under these different conditions, permitting comparisons of results. All experiments presented focused on partially expanded leaf 8 (counting the first seedling leaf as leaf 1) when it was 50–60 cm long with a sheath <1 cm long (unexpanded).

### Cell area measurements

Leaf 8 was cut into 2-cm segments, stained for 10–15 min with 10 µg mL^−1^ propidium iodide, washed in water, and mounted on glass slides in water under a coverslip. Pavement cell outlines were visualized using a previously described (Walker *et al.*, 2007) confocal microscope system with 514 nm excitation from an argon gas laser and a 570/65 nm bandpass emission filter. Surface areas were measured on a Macintosh computer using the public domain NIH Image program (developed at the US National Institutes of Health and available at http://rsb.info.nih.gov/nih-image/; Schneider *et al.*, 2012). Three fields of view were analysed at each leaf stage for each of three individual leaves (*n* > 130 cell areas measured at each position along the developmental gradient).

### Cuticle permeability experiments

Intact B73 leaf 8 was excised, the cut end was sealed with petroleum jelly, and the leaf was submerged in 0.05 % Toluidine Blue O (TBO) in water (Tanaka *et al.*, 2004). After 1 or 2 h, leaves were photographed and successive 1-cm segments were homogenized and extracted with isopropanol/formic acid. To separate TBO from chlorophyll, extracts were treated with hexane. TBO in the aqueous phase was quantified by spectrophotometry (A630 nm). To measure resistance to dehydration, intact plants were kept in the dark for 2 h to close stomata, so that water was lost mainly by transpiration across the cuticle (Ristic and Jenks, 2002). To measure water loss, 2-cm segments were photographed and weighed repeatedly over a 2-h period. Weight loss was normalized against surface areas determined from photographs using Image J (developed at the US National Institutes of Health and available at http://rsb.info.nih.gov/nih-image/Schneider *et al.*, 2012).

### Transmission electron microscopy

Samples were prepared for TEM following previously described procedures (Wilson and Bacic, 2012; Guzmán *et al.*, 2014) with minor modifications. Leaf tissue pieces (~2 mm × 10 mm) were cut from the area midway between the midrib and leaf margin within each developmental interval analysed, and fixed in 2 % paraformaldehyde/2 % glutaraldehyde in 0.1 m sodium cacodylate buffer (pH 7.3–7.5) for 3 d under vacuum at 4 °C. Samples were washed with 0.1 m sodium cacodylate buffer (×4) and postfixed in 1:1 solution of 2 % osmium tetroxide in 0.1 m sodium cacodylate for at least 12 h at 4 °C. Samples were washed with 0.1 m sodium cacodylate buffer (×2) and then water (×2) before second postfixation in aqueous 2 % uranyl acetate overnight at 4 °C. Samples were washed with water (×2) before passing through an acetone dehydration series (20, 40, 60, 80 and 100 %, 30 min each) on ice and finally 100 % dry acetone at room temperature. Samples were infiltrated in an acetone:epoxy resin (Durcupan ACM, Sigma) series (v/v, 3:1 2 h, 1:1 overnight, 1:3 2 h, 0:1 overnight) and embedded in resin at 60 °C for at least 3 d. Seventy-nanometre sections were cut using a Leica UCT ultramicrotome and collected on formvar/carbon copper 100-mesh grids (Electron Microscopy Sciences) with glow discharge and post-stained with aqueous 2 % uranyl acetate and Reynolds lead citrate for 5 min each. Grids were viewed on a JEOL JEM-1400Plus (80 kV) TEM equipped with a Gatan OneView camera or with a Tecnai G2 Spirit BioTWIN (80 kV) TEM equipped with an Eagle 4k HS digital camera (FEI, Hilsboro, OR).

### Cuticular lipid analysis

#### Cuticular wax extraction: optimization of wax extraction method

Cuticular waxes are, by definition, organic-solvent-soluble, while cutin is insoluble (Kolattukudy, 1980). Most plant surface waxes are efficiently extracted by quick immersion in chloroform (Holloway, 1984; Riederer and Schneider, 1990; Jetter *et al.*, 2006; Fernandez-Moreno *et al.*, 2016; Hegebarth and Jetter, 2017). However, it is unclear if other solvents or combinations of solvents that are more or less polar than chloroform can equally extract the main wax classes identified in maize mature leaf samples. Thus, wax extraction conditions were first optimized for solvent polarity and extraction time (Loneman *et al.*, 2017). A portion of the partially expanded leaf 8 between 18 and 24 cm from the leaf base was used for all extractions. Three solvent systems were compared: hexane:diethyl ether (9:1) [2.12], chloroform:hexane (1:1) [3.35], and pure chloroform [4.81], listed in order of increasing polarity (dielectric constants are shown in square brackets). Leaf segments were immersed in each solvent and gently shaken for 1 min. To further optimize the immersion time, tissues were extracted with chloroform for 0.5, 1, 2 and 5 min. Because no significant differences were observed with any of the solvents or extraction times analysed (Supplementary data Fig. S1), dipping in chloroform for 1 min was used for all subsequent wax determinations.

#### Wax extraction from developing leaves

Pieces 2 cm long (excised between 2 and 22 cm from the leaf base) were immersed in 5 mL of chloroform and 5 μg of each internal standard, namely *n*-tetracosane (24:0 alkane), 1-pentadecanol (15:0-OH) and heptadecanoic acid (17:0), was added to each extract. The chloroform extracts were evaporated under a nitrogen stream. Each leaf portion was scanned and its surface area measured using ImageJ and multiplied by 2 to account for both surfaces.

#### Isolation of epicuticular and intracuticular waxes

Epicuticular waxes were removed by applying a gum arabic solution on the surface (Jetter and Schaffer, 2001). Intracuticular waxes were calculated by subtracting epicuticular waxes from total chloroform-extracted cuticular waxes. Gum arabic (Acros Organics) was washed with chloroform using a Soxhlet extractor at 40 °C for 48 h (Buschhaus and Jetter, 2007) and subsequently used to prepare a 0.6 g mL^−1^ solution in sterile deionized water. Twelve plants were employed to produce four biological replicates per sample. After removing the midrib, one half of each leaf segment was used to extract epicuticular waxes and the other half was immersed in chloroform for total cuticular wax analysis. Gum arabic solution was applied to four 3-cm-long segments corresponding to 4–7, 8–11, 12–15 and 19–22 cm from the leaf base; for each sample, the gum was applied to two pieces on the adaxial side and two pieces on the abaxial side. Gum pieces were peeled off and photographed alongside a ruler to determine surface area using ImageJ software. Gum pieces were extracted with chloroform and waxes analysed following the same procedure described above.

#### GC–*flame ionization detection* and GC*–*MS analysis

Wax extracts were transformed into their trimethylsilyl ester and ether derivatives following the protocol described by Razeq *et al.* (2014). For chemical identifications, the samples were analysed by GC–MS on a TRACE 1300 Thermo Scientific GC with a Thermo Scientific ISQ Single Quadrupole MS detector. Splitless injection was used with a VT-5HT capillary column (30 m × 0.25 mm internal diameter and 0.10 µm film thickness) and a helium flow set at 1.5 mL min^−1^. Temperature settings were as follows: inlet 330 °C; detector 300 °C; oven temperature set at 150 °C for 3 min and then increased to 330 °C at a rate of 3 °C min^−1^, then from 330 °C to 390 °C at 6 °C min^−1^, with a final hold at 390 °C for 10 min. For compound quantification, wax samples were analysed on a Thermo Scientific TRACE 1300 gas chromatograph coupled to a flame ionization detector (GC–FID) using column and chromatographic conditions similar to those described for the GC–MS analysis. Wax components were identified by their relative retention times and characteristic mass spectra by comparison with published MS data (e.g. Christie, 2009) or by searching a mass spectral reference library (NIST 2011). The approach used to determine the double bond position of the alkenes identified in our analysis is described in Supplementary data Method S1. Wax components were quantified based on the total FID ion current. Theoretical correction factors were applied that assume that the FID response is proportional to carbon mass for all carbons bonded to at least one H atom (Christie, 1991). Because split/splitless injection is known to bias against high-molecular-weight compounds, calibration curves for wax esters with even-chain standards were generated and used to calculate the amounts of wax esters found in the samples (Supplementary data Method S2, Fig. S2).

### Lipid polyester analysis

Leaf segments used for wax extractions were delipidated by shaking in isopropanol and chloroform:methanol mixtures, as described in Jenkin and Molina (2015). Polyester monomers released by transesterification of solvent-extracted tissues were transformed into silylated derivatives and analysed by GC–MS (Jenkin and Molina, 2015). Cutin monomers were identified by their relative retention times and characteristic fragmentation patterns, and by comparison of the electron ionization mass spectra of their trimethylsilyl derivatives with published spectra (Eglington and Hunneman, 1968; Eglinton *et al*., 1968; Holloway and Deas, 1971; Holloway and Deas, 1973). Control experiments to assess the monomer contribution by other leaf tissues (i.e. bundle sheath suberin) are detailed in Supplementary data Method S3.

### Statistical analyses

Statistical analyses were conducted with GraphPad Prism v6.0, using one-way ANOVA with Tukey’s multiple comparison post-test.

## RESULTS

### The adult maize leaf cuticle acquires its water barrier property at the cessation of cell expansion

**Fig. 1.**
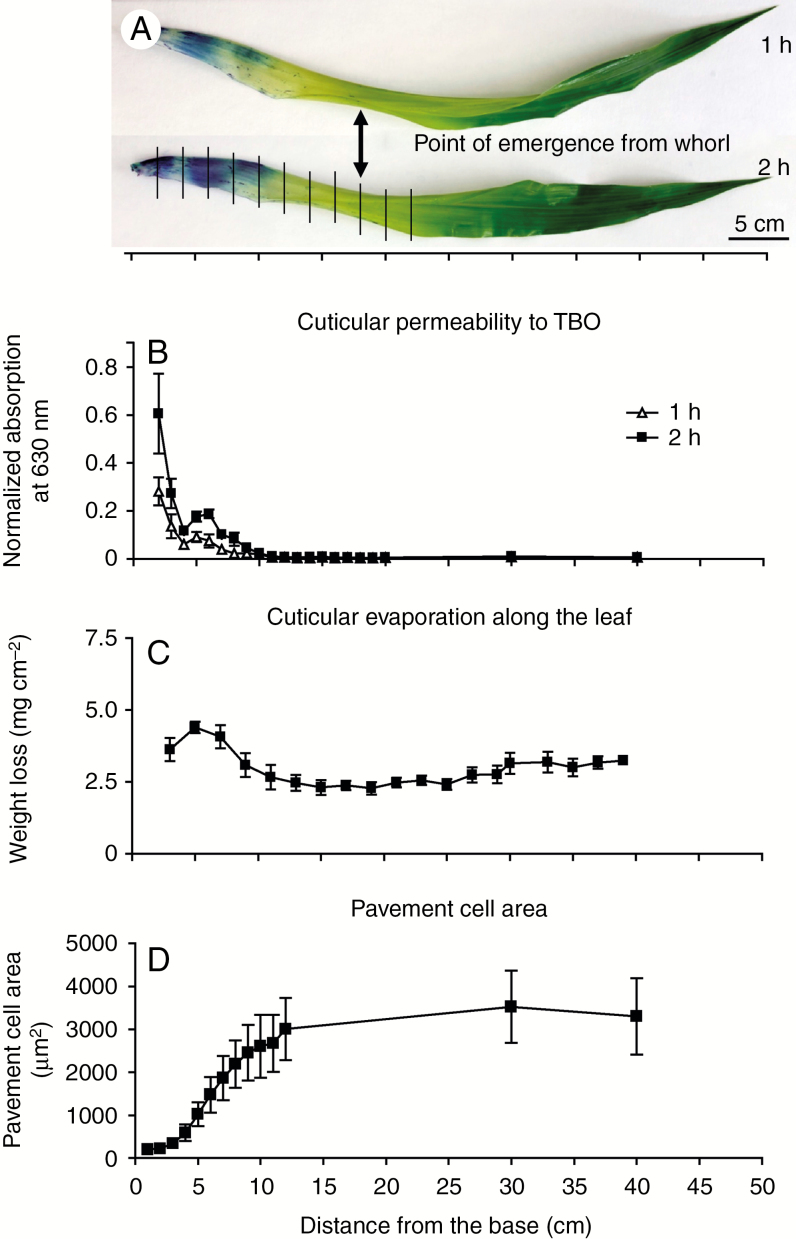
Cuticle maturation along the developmental gradient of a partially expanded, adult maize leaf. (A) Intact adult maize leaves (B73, leaf 8 at 50–60 cm length) stained with TBO for 1 or 2 h. Vertical lines indicate segments harvested for subsequent analysis of cuticle composition and ultrastructure. (B) Quantification of TBO penetration of the cuticle. Results at each position are expressed as absorbance readings at 630 nm normalized to surface area. (C) Cuticular evaporation rate, a measure of dehydration resistance, was measured for successive 2-cm segments. (D) Pavement cell surface areas. In (B–D), *n* = 3 leaves per time point, mean ± s.e.

The maize leaf develops from tip to base in a continuous gradient, providing an excellent system for comparative analysis of different developmental stages in a single leaf at one time point (Sylvester and Smith, 2009). For this study we chose leaf 8, the first fully adult leaf, in a partially expanded state (50–60 cm long) of the inbred line B73 as the standard for all experiments. Cells are still dividing at the base of this leaf, with later developmental stages (cell expansion, differentiation, maturation) represented at successively more distal positions (Facette *et al.*, 2013). To investigate the timing of water barrier establishment along this gradient (Fig. 1), we first evaluated permeability to the water-soluble dye TBO. This dye is a hydrophilic molecule larger than water, but prior work has established that leaves of cuticle mutants in *Arabidopsis* have increased permeability to TBO (Tanaka *et al.*, 2004) and TBO penetration has been used previously to track cuticle maturation in developing leaves (Richardson *et al.*, 2007; Hachez *et al.*, 2008). Toluidine Blue O penetrated the developing cuticle until ~10–12 cm above the leaf base (Fig. 1A, B). In a second approach, we found that dehydration rates of leaf segments kept in the dark to close stomata (though some water could still be lost through immature or incompletely sealed stomatal pores) also decreased gradually from the leaf base to ~10–12 cm (Fig. 1C). Though neither method used directly measures water transport rates across the cuticle, they were in close agreement in localizing the point along the leaf gradient where minimal values are reached as 10–12 cm from the leaf base. To relate these findings to cell expansion, we measured pavement cell areas along the developmental gradient. Almost all cell elongation occurred between 3 and 12 cm from the leaf base, with the highest cell expansion rate between 4 and 8 cm (Fig. 1D), where there is a local increase in TBO permeability (Fig. 1A, B). Together, these results indicate that the water barrier property of the adult maize leaf cuticle becomes fully established ~10 cm above the leaf base, coinciding with the completion of cell elongation and shortly before emergence of the leaf from the whorl (point of emergence, at 17–18 cm from leaf base; Fig. 1A).

### The timing of accumulation of different wax types and chain lengths varies widely along the adult maize leaf developmental gradient

**Fig. 2.**
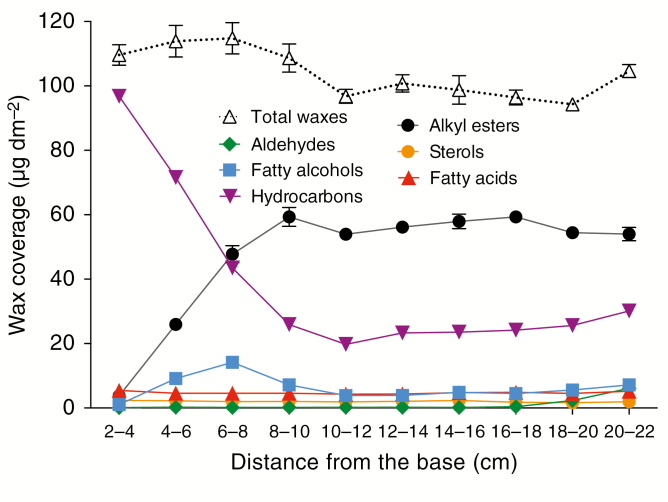
Cuticular wax composition along the adult maize leaf developmental gradient. Changes in the accumulation of six classes of compounds present in chloroform-extracted wax mixtures (based on data shown in Supplementary data Table S1). Mean of four replicates and s.e. are reported.

To elucidate the compositional changes associated with cuticle ontogeny in adult maize leaves, we analysed both cuticular waxes and cutin along this developmental gradient. Given the immaturity of the cuticle between 2 and 4 cm from the leaf base, wax loads at this position were surprisingly high (109 ± 6 μg dm^−2^) compared with the total wax coverage of the more mature portions analysed. Overall wax load did not increase across the developmental gradient, with amounts fluctuating between 94 and 115 μg dm^−2^ (Fig. 2). However, wax composition varied dramatically across the gradient. Hydrocarbon (alkane/alkene) coverage was highest 2–4 cm from the leaf base, decreasing by 70 % from there to 10–12 cm (Fig. 2; Supplementary data Table S1). Alkyl esters followed the opposite trend, accumulating from nearly undetectable levels initially to become the most prevalent class of waxes by the 10- to 12-cm stage, when the water barrier property is fully established, reaching a coverage of ~59 μg dm^−2^ at this position and remaining at this level thereafter. Fatty alcohols reached their highest concentration between 6 and 8 cm from the leaf base (14.2 μg dm^−2^) and subsequently decreased, possibly because alcohols are incorporated into wax esters. Free fatty acids, aldehydes and alicyclics represented a small fraction of waxes at all stages and did not change greatly in abundance across the developmental time-course analysed (Fig. 2; Supplementary data Table S1).

**Fig. 3.**
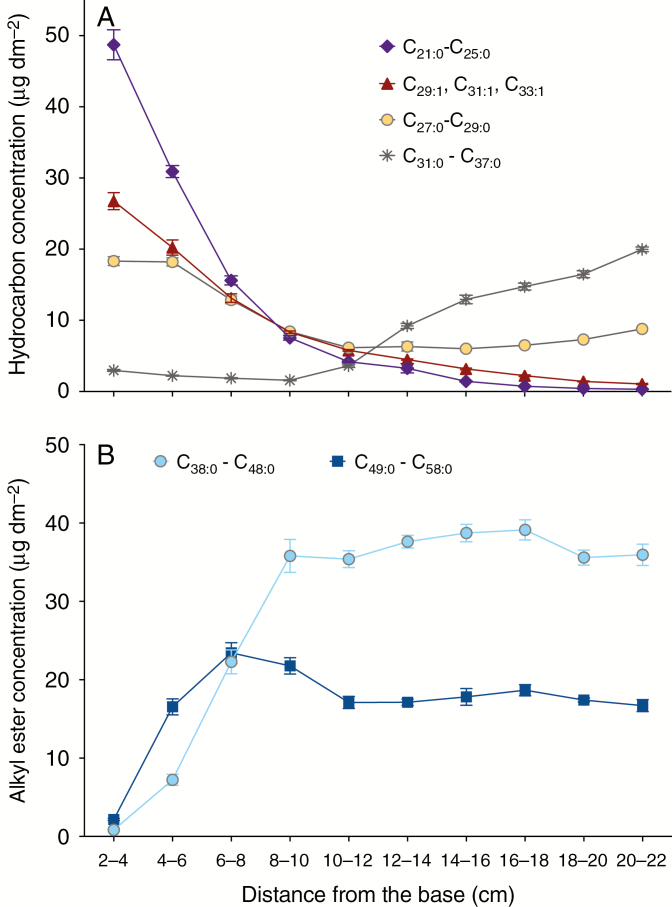
Hydrocarbon and alkyl ester composition along the adult maize leaf developmental gradient. (A) Hydrocarbon (alkane and alkene) composition. (B) Alkyl ester composition (low-abundance components, namely C_38_ and odd-carbon-number esters, are not shown). Mean of four replicates and s.e. are reported.

Conspicuous compositional changes were observed within the hydrocarbon series. C_21:0_, C_23:0_ and C_25:0_*n*-alkanes, as well as C_29:1_ and C_31:1_ alkenes, were the predominant hydrocarbons at the earliest stages analysed. However, these components decreased in abundance gradually across the gradient, reaching low abundance by the 10- to 12-cm stage, when the water barrier property is fully established, and nearly disappearing thereafter (Fig. 3A; Supplementary data Table S1). Each C_29:1_ and C_31:1_ alkene included two positional isomers – determined by MS analysis of their corresponding dimethyl disulphide adducts (Buser *et al.*, 1983) – namely 9- and 10-nonacosene and 9- and 10-hentriacontene (Supplementary data Fig. S3). C_27:0_ and C_29:0_*n*-alkanes also declined in abundance from 2 to 12 cm, remaining constant thereafter. The longest *n*-alkanes (C_31:0_ to C_37:0_) were almost undetectable until the 8- to 10-cm stage, and also increased in abundance thereafter. Thus, we observed an overall shift from shorter to longer hydrocarbons as development proceeded, with chain lengths of <30 carbons predominating initially but shifting to >30 carbons by the time cuticle development was complete.

Although alkanes were the dominant class 2–8 cm from the base, alkyl esters were the most prevalent group of waxes beyond this point (Fig. 2; Supplementary data Table S1). Two groups of wax esters were found, one containing the C_38_–C_48_ homologues (including small proportions of odd-carbon-numbered species), and a second composed mostly of C_50_–C_58_ homologues. The distribution and amount of these two groups differed along the gradient (Fig. 3B). In contrast to findings for alkanes, the longer alkyl esters accumulated first, reaching their peak abundance by 6–8 cm and declining slightly thereafter, while the shorter C_38_–C_48_ group of alkyl esters reached nearly peak levels by the 8- to 10-cm stage with little or no further changes thereafter (Fig. 3b; Supplementary data Table S1). Furthermore, this shorter-chain alkyl ester group accumulated to approximately twice the abundance of the longer-chain group (Fig. 3B). Each alkyl ester class was composed of a mixture of isomers. Overall, the acid and alcohol moieties 20–22 cm from the leaf base were 18:0–22:0 (92 % of the esterified fatty acids) and 22:0–34:0 (86 % of the esterified alcohols), respectively (Supplementary data Table S2).

The composition of the free fatty alcohol fraction (i.e. primary alcohols not incorporated into alkyl esters) reflected that of the alcohols that are incorporated into alkyl esters (Supplementary data Tables S1 and S2); only 1-docosanol was not found in its free form. In spite of acyl chains varying between 16 and 30 carbons in length in the alkyl esters, only 16:0 and 18:0 free fatty acids were detected in the wax mixtures of any stage analysed (Supplementary data Table S1). Collectively, the broad chain-length distribution of wax ester homologues and their isomers and the fact that the bulk of primary alcohols and fatty acids are incorporated into wax esters suggests that one or more wax ester synthases efficiently incorporate substrates of various chain lengths.

The aldehyde fraction comprised an even-numbered homologous series ranging from 26 to 34 carbons in length (Supplementary data Table S1). Unlike the other wax classes, aldehydes only accumulated significantly >18 cm from the base, with an overall amount of only 5.6 % of the total waxes in the segment occurring between 6 and 22 cm from the leaf base.

Alicyclic triterpene derivatives identified in the analysed leaf 8 developmental stages largely comprised sterols, including β-sitosterol, campesterol and stigmasterol (Supplementary data Table S1). Neither the overall amount nor the relative proportions of sterols varied substantially across the developmental time-course analysed (Supplementary data Fig. S4). Thus, variation in alicyclic wax content does not appear to play a role in establishing the water barrier property of cuticle during adult maize leaf development.

### Cutin and wax deposition along the maize leaf developmental gradient is not synchronized

**Fig. 4.**
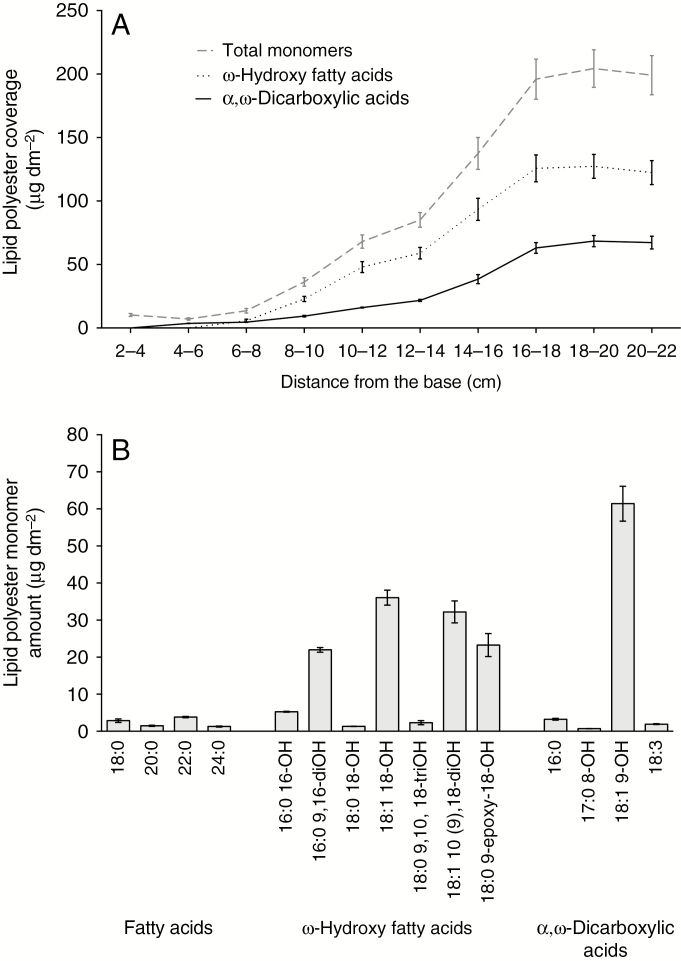
Lipid polyester monomer accumulation along the adult maize leaf developmental gradient. (A) Accumulation of the two main classes of cutin monomers present in maize leaf cutin compared with the total amount of monomers. (B) Representative profile of the maize mature leaf cutin monomer composition (portion between 20 and 22 cm from the leaf base). Means of four replicates and s.e. are reported.

Unlike waxes, cutin steadily increased in abundance from the 4- to 6-cm segment to the 16- to 18-cm segment of the leaf developmental gradient, reaching a surface mass per unit area twice that of the combined waxes and remaining constant thereafter (Fig. 4A). Another notable difference compared with wax biogenesis was that the relative proportions of the main two classes of cutin monomers identified, ω-hydroxy acids and α,ω-dicarboxylic acids, remained constant at about 2:1 as both accumulated (Fig. 4A). Largely consistent with an earlier study of cutin composition in adult maize leaves of a different inbred line (Espelie and Kolattukudy, 1979), the major cutin monomers in adult B73 leaves were identified as 9,16-dihydroxyhexadecanoic acid, 18-hydroxyoctadecenoic, 10(9),18-dihydroxyoctadecenoic acid, 9-epoxy-18-hydroxystearic acid and 9-hydroxy-1,18-octadecene dioic acid, which together accounted for 86 mole % of the cutin load at maturity (Fig. 4B; Supplementary data Table S3). Two of these monomers, 18:1 10(9),18-dihydroxyoctadecenoic acid and 9-hydroxy-1,18-octadecene dioic acid, have been found previously in the depolymerization products of maize leaf cutin (Holloway, 1983). However, these monomers are unlikely to be products of enzyme-catalysed reactions; instead, they probably result from photo-oxidation and auto-oxidation of unsaturated fatty acids (Kosma *et al.*, 2015). Although aromatics (i.e. ferulic and coumaric acids) were abundant components of the cutin profiles in our analysis utilizing whole leaves, analysis of cutin monomers in enzymatically isolated epidermal tissues indicated that the bulk of these hydroxycinnamic acid derivatives, along with hexadecanoic acid, are of non-epidermal origin (Supplementary data Fig. S5). Therefore, the cuticle composition results reported here do not include ferulate, coumarate and hexadecanoic acid (Supplementary data Table S3).

### Ultrastructural analysis of pavement cell cuticles in the adult maize leaf reveals distinct layers but no cuticular layer

**Fig. 5.**
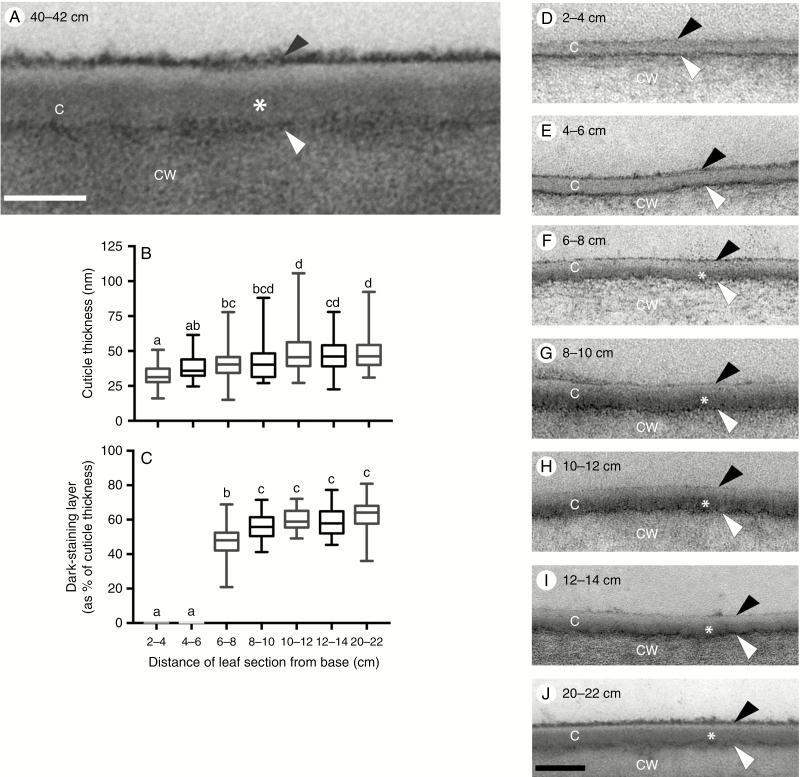
B73 leaf pavement cell cuticle development visualized by TEM. (A) Pavement cell cuticle from a partially expanded leaf 8, 40–42 cm from the base, where leaf tissue is mature. Four distinct layers or zones are visible: a thin, darkly stained layer (white arrowhead) at the interface between the cell wall (CW) and cuticle, dark (asterisk) and light zones of the cuticle proper (C), and a darkly stained epicuticular layer (black arrowhead). Scale bar = 40 nm. (B) Thickness of pavement cell cuticles at the indicated positions along the developmental gradient of partially expanded B73 leaf 8. (C) Percentage of cuticle thickness at indicated positions occupied by the dark-staining inner layer of the cuticle proper. In (B) and (C), lower-case letters indicate significance groups identified by one-way ANOVA with the Tukey multiple comparisons post-test. (D–J) Representative images of pavement cell cuticles at the indicated positions along the developmental gradient of partially expanded B73 leaf 8. Scale bar = 100 nm.

**Fig. 6.**
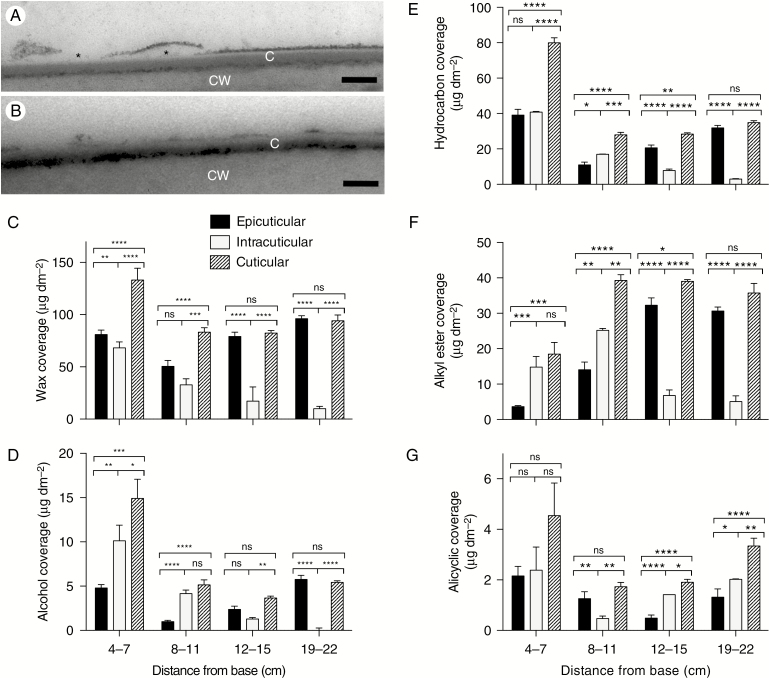
Isolation and analysis of the epicuticular wax layer. (A) Mature adult maize leaf showing that the dark-staining external layer of the cuticle becomes detached in places (asterisks), demonstrating a loose association of this layer with the most internal part of the cuticle. (B) Stripping a mature adult maize leaf with gum arabic removes most of the dark-staining external layer. C, cuticle; CW, cell wall. Scale bars (A, B) = 100 nm. (C–G) Epicuticular, intracuticular and total wax composition in four segments, between 4 and 22 cm from the base, of the maize leaf 8 developmental gradient. Epicuticular waxes removed by adhesion to, and subsequent chloroform extraction from, gum arabic were compared with total wax extracted with chloroform from a matched sample; intracuticular waxes were inferred by calculating the difference between epicuticular and total waxes. Total wax coverage (C) and more abundant wax classes, namely alcohols (D), hydrocarbons (E), alkyl esters (F) and alicyclics (G), are shown for each fraction. Means of three replicates for chloroform-extracted waxes and of six replicates (from three adaxial and three abaxial) for epicuticular waxes and s.e. are reported. *P < 0.05; **P < 0.01; ***P < 0.001; ****P < 0.0001; ns, not significant.

Prior studies of maize leaf cuticle ultrastructure have examined only juvenile leaves, and the published images and reports do not reveal many ultrastructural details (Holloway, 1982; Ristic and Jenks, 2002; Sturaro *et al.*, 2005). Ultrastructural images of adult maize leaf pavement cells, revealed a thin cuticle (~40 nm) composed of four zones with distinct osmium staining characteristics (Fig. 5A). The boundary between the cell wall and cuticle is demarcated by a darkly stained interface (white arrowhead in Fig. 5A), which presumably corresponds to the pectin-rich wall/cuticle interface described for many other plant species (Jeffree, 2006). Another darkly stained layer (black arrowhead in Fig. 5A) was observed at the outer surface of the adult maize leaf cuticle. This layer was loosely associated with the rest of the cuticle and was removable with gum arabic (Fig. 6A, B), identifying it as epicuticular wax. Between the wall/cuticle interface and the epicuticular layer, darker (asterisk) and lighter-staining zones were observed (Fig. 5A). The lack of fibrils indicates that the darker zone should be classified as a layer of the cuticle proper rather than as a cuticular layer. However, cuticles of specialized cell types (bulliform cells, stomatal guard and subsidiary cells) in the adult maize leaf epidermis were much thicker and exhibited a cuticular layer (Supplementary data Fig. S6).

We next examined the developmental origins of mature leaf cuticle ultrastructure, by TEM imaging of pavement cell cuticles at a series of 2-cm intervals along the developmental gradient of a partially expanded adult maize leaf 8. After finding no ultrastructural differences between adaxial and abaxial pavement cell cuticles in the 20- to 22-cm interval (Supplementary data Fig. S7), we did not attempt to distinguish adaxial and abaxial cuticles in earlier intervals. Cuticle thickness increased by 33 % across our developmental gradient, from ~30 to 40 nm and reaching this final thickness ~10 cm from the leaf base (Fig. 5B). At the youngest intervals examined (2–4 and 4–6 cm from the leaf base), both the wall/cuticle interface and the epicuticular layer were already present; however, the cuticle proper was not yet differentiated into darker and lighter zones (Fig. 5D, E). The darker zone of the cuticle proper first appeared 6–8 cm from the leaf base (asterisk in Fig. 5F), reaching its maximum thickness (about two-thirds of the thickness of the entire cuticle) ~10 cm from the leaf base (Fig. 5C, G–J). Prior studies of cuticle ontogeny have consistently shown that dark- and light-staining zones within the cuticle proper emerge during cell expansion (which in our system is completed within 10–12 cm of the leaf base), whereas the cuticular layer emerges later, after completion of cell expansion, in concert with substantial deposition of cutin (e.g. Riederer and Schönherr, 1988; reviewed by Jeffree, 2006). We observed no significant changes in the thickness or appearance of any cuticle zones or layers, or of the cuticle as a whole, beyond the 10- to 12-cm interval (Fig. 5B, C, G–J), supporting the conclusion that adult maize leaf pavement cells have no distinguishable cuticular layer when observed by TEM. Thus, the ultrastructural features of the mature pavement cell cuticle are established at the same time that the cuticle as a whole reaches functional maturity as a barrier to hydrophilic molecules and cell expansion is completed.

### Most cuticular wax of the mature adult maize leaf is epicuticular

To compare the composition of epicuticular and intracuticular waxes and how they change during cuticle ontogeny, epicuticular waxes were mechanically removed with gum arabic from both adaxial and abaxial surfaces (Jetter and Schaffer, 2001) and extracted from gum arabic with chloroform. Epicuticular waxes recovered in this way were compared with total chloroform-extractable cuticular wax; the difference was considered to represent intracuticular wax. For this experiment, 3-cm-long segments of the leaf 8 gradient were analysed at 4–7, 8–11, 12–15 and 19–22 cm from the base (Fig. 6C–G). This analysis showed a similar abundance of total waxes (and hydrocarbons, the most abundant wax type at this stage) in both layers between 4 and 11 cm (Fig. 6C, E), with a subsequent increase in the relative abundance of epicuticular waxes. By 19–22 cm from the leaf base, the vast majority of extractable waxes were found in the epicuticular fraction (Fig. 6C–F). Alicyclic waxes were found in both epicuticular and intracuticular fractions at all stages analysed, with similar abundance in both fractions at maturity (19–22 cm from the leaf base; Fig. 6G). Consideration of these results in relation to those presented earlier suggests that the bulk of the ultrastructurally defined cuticle proper consists of wax (predominantly hydrocarbons) at the earliest developmental stages analysed, but at maturity it is composed mostly of cutin. Thus, functional maturation of the cuticle as a water barrier is associated with a decrease in the amount of intracuticular wax as well as a compositional shift in its wax content concomitant with an increase in cutin.

## DISCUSSION

The cuticle protects shoot tissues from water loss and is thus a promising target for improving drought tolerance. Cuticle modification through breeding or transgenic strategies is hampered by lack of knowledge of what compositional or structural characteristics of the cuticle are most important for its water barrier properties, and to what extent the determinants are species- or tissue-specific. In this study, we investigated cuticle features related to water permeability by comparing cuticle composition, ultrastructure and water barrier properties at a series of stages of maize leaf development. Our study is one of the first to characterize cuticles of adult maize leaves, the leaf type determining most of the agronomically significant leaf-related traits in maize.

The development of monocot leaves in a tip-to-base gradient where the youngest tissues are tightly wrapped inside a whorl of older leaves protecting them from desiccation, while older tissues are exposed to the air, provides a unique opportunity to query the relationship between cuticle biogenesis and its acquisition of water barrier properties. Indeed, the use of developing monocot leaves to study structural and/or compositional changes in cuticle lipids during development was established >40 years ago (Giese, 1975; Avato *et al.*, 1980). Consistent with prior studies focused on seedling leaves of barley (Richardson *et al.*, 2007) and maize (Hachez *et al.*, 2008), we found that the water barrier property of developing adult maize leaves is established around the time that cell expansion is completed (~10 cm from the leaf base), well before leaves emerge from the whorl (the ‘point of emergence’, ~17 cm from the leaf base). However, in contrast to findings for developing leaves of barley(Richardson *et al.*, 2007) and leek (Rhee *et al.*, 1998), where little or no cuticular wax was detected prior to cessation of cell expansion, we found that waxes are already abundant at the earliest stages analysed in developing maize leaves, where they might have a role in maintaining organ separation, although this function is generally attributed to cutin (reviewed by Fich *et al.*, 2016). Our results also contrast with those reported for inbred B73 silk waxes, where 3-fold more hydrocarbons are present on emerged silks compared with encased silk portions (Perera *et al.*, 2010). Thus, there seems to be variation between species, leaf types and/or tissue types with respect to the timing of wax deposition during development of monocot organs developing in a tip-to-base gradient.

In spite of the near-constant overall abundance of waxes, wax composition varies dramatically across the adult maize leaf developmental gradient. At 2–4 cm from the leaf base, waxes were composed almost entirely of alkanes and alkenes, which gradually decreased to ~20 % of total wax by the time the water barrier was established at ~10 cm. Concomitantly, wax esters increased from nearly undetectable levels initially to become the predominant wax class (~60 % of the total) at ~10 cm. Fatty alcohols, free fatty acids and aldehydes remained minor components throughout the developmental period analysed.

Our results for mature adult maize leaf wax composition are in broad agreement with an earlier study (Bianchi *et al.*, 1984) and show considerable differences from juvenile leaves of maize (e.g. Bianchi *et al.*, 1978) and seedling leaves of barley (Richardson *et al.*, 2005), where waxes are dominated by fatty alcohols and aldehydes, with low proportions of alkanes and esters. Moreover, since previously published scanning electron microscopy images reveal an absence of epicuticular wax crystals on adult maize leaf surfaces under conditions where they are observed on juvenile leaves (Sylvester *et al.*, 1990; Yang *et al.*, 1993; Sylvester and Smith, 2009), we conclude that the epicuticular wax layer we observed on adult leaf surfaces via TEM consists of an amorphous film without crystalline structure. While waxes (particularly fatty alcohols and wax esters) are predominantly intracuticular before and during establishment of the water barrier (4–7 and 8–11 cm from the leaf base), at later stages almost all extractable waxes are epicuticular. Maize appears to be unusual in this regard, since most studies comparing epi- and intracuticular waxes in mature leaves of various dicot species show substantial proportions of wax in the intracuticular fraction (e.g. Jetter and Riederer, 2016; Zeisler-Diehl *et al.*, 2018). It is unclear whether this is an unusual characteristic of maize leaf waxes or whether growth chamber conditions may affect the proportion of waxes removed by gum arabic. Although the composition of epicuticular wax changes considerably over the time-course of adult maize leaf development, its appearance in TEM does not vary. This is consistent with the expectation that saturated aliphatic molecules, whose relative proportions in epicuticular wax are changing, do not react with osmium tetroxide (Cheng *et al.*, 2009; Shumborski *et al.*, 2016). The osmiophilic character of the epicuticular wax film we observe via TEM, which has been observed on leaf surfaces of a variety of other species, such as *Hedera helix* (Viougeas *et al.*, 1995) and *Clivia miniata* (Merida *et al.*, 1981), may reflect the presence of unsaturated alicyclic molecules present at the developmental stages analysed, predominantly sterols.

Within the alkane homologue series, we observed a dramatic shift in the distribution of chain lengths over the course of cuticle ontogeny: the blend dominated by C_21_ and C_23_ alkanes 2–4 cm from the leaf base gradually shifts to one dominated by ≥29-carbon alkanes at the point of leaf emergence from the whorl (16–18 cm), remaining similar thereafter. This shift is achieved via reductions in the amounts of most alkanes between 6 and 12 cm from the leaf base (likely resulting from synthesis not keeping up with the rapid cell expansion occurring in this interval) followed by deposition of C_29_–C_37_ alkanes after completion of cell expansion. Shifts from shorter to longer hydrocarbon chain lengths have been previously reported as a feature of cuticle maturation in dicot leaves (Jenks *et al.*, 1996; Jetter and Schaffer, 2001; Busta *et al.*, 2017) and in development of the fifth leaf, a juvenile leaf in maize (Avato *et al.*, 1980). In developing *Arabidopsis* leaves, this shift is associated with an increase in the expression level of CER6, a fatty acid elongase needed to produce acyl chains longer than 28 carbons *in vitro* and *in vivo* (Busta *et al.*, 2017, Haslam *et al.*, 2012). The functional significance of the shift towards longer hydrocarbon chain lengths is unclear and not apparently related to establishment of the cuticular water barrier property in *Arabidopsis* leaves, which are already exposed to the air prior to the shift. Similarly in maize, we found that accumulation of alkanes with chain lengths longer than 30 carbons occurred after establishment of the water barrier in adult maize leaf cuticles at ~10 cm from the leaf base.

Among the wax compositional changes we observed across the time-course of adult maize leaf development, only the timing of wax ester accumulation correlates with establishment of the water barrier property of the adult maize leaf cuticle. Thus, our findings suggest a possible role for wax esters in protecting adult maize leaves from dehydration. Consistent with this conclusion, recent work has demonstrated that although wax esters are a very minor component of cuticular waxes in *Arabidopsis*, reduction of this wax type in *wsd1* wax ester synthase mutants increases drought sensitivity and cuticular permeability (Patwari *et al.*, 2019). However, it is unclear whether these phenotypic effects reflect a key role for wax esters in cuticular impermeability, or are due to changes in stomatal density, also observed in *wsd1* mutants. The structure of wax esters consisting of two long hydrocarbon molecules linked by a central ester bond might facilitate a parallel arrangement of hydrocarbon tails that can become organized into crystalline domains within the cuticle that efficiently exclude water (Riederer and Schreiber, 2001; Buschhaus and Jetter, 2012). However, wax ester abundance across the maize leaf developmental time-course increases only in the epicuticular fraction, while decreasing in the intracuticular fraction. Epicuticular wax has been found to play a minor role, if any, in cuticle impermeability to water (Jetter and Riederer, 2016; Zeisler-Diehl *et al.*, 2018). The role of wax esters in resistance to dehydration in adult maize leaves and the relative contributions of epi- versus intracuticular wax esters remain to be investigated.

We found that cutin is present at very low abundance 2–6 cm from the leaf base, gradually increasing in abundance from there to the 16- to 18-cm interval, where leaves emerge from the whorl and are exposed to the air. Unlike the shifts in wax composition observed along the adult maize leaf developmental gradient, we observed a relatively constant ratio between the major cutin monomers at all stages analysed (~2:1 ratio of ω- hydroxy fatty acids to α,ω-dicarboxylic acids); however, it is not possible to determine whether this reflects homogeneity in cutin architecture over time. Our findings contrast with those for developing juvenile barley leaves, where no further cutin deposition was observed beyond the point where cell expansion was completed (Richardson *et al.*, 2007) but are consistent with findings for developing leaves of *Clivia miniata* (a monocot), where cutin deposition continued well beyond the cessation of cell expansion (Riederer and Schönherr, 1988). In contrast to *C. miniata*, where an increase in cuticle thickness parallels the accumulation of cutin, we observed no increase in pavement cell cuticle thickness beyond the 8- to 10-cm stage. Notably, bulliform and stomatal cells in adult maize leaves have much thicker cuticles. Consequently, the increase in cutin we observe from 10 to 22 cm from the leaf base is likely due to selective accumulation of cutin in the cuticles of those cells or other specialized cells we did not analyse, such as macrohairs.

While prior studies have described cuticle ontogeny in ultrastructural terms and/or in relation to lipid composition (e.g. Riederer and Schönherr, 1988), few have attempted to relate these developmental events to the functional maturation of the cuticle. Among the ultrastructural changes we observed across the time-course of adult maize leaf development, the emergence of a dark-staining inner zone of the cuticle proper coincides with the establishment of the water barrier property, appearing initially 6–8 cm from the leaf base and reaching its final thickness and appearance at 8–10 cm. While the emergence of this layer coincides with wax ester accumulation in the cuticle as a whole, wax esters are not osmiophilic and accumulate largely in the epicuticular fraction. The emergence of the dark-staining inner layer of the cuticle proper more likely reflects the deposition of cutin. While cutin alone is not an effective water barrier, it is thought to provide a scaffold for the deposition of waxes needed to form a functional cuticle. Thus, we hypothesize that although intracuticular waxes are depleted as the cuticle matures, an association between cutin and the remaining intracuticular waxes that is established initially between 6 and 10 cm from the leaf base is a key feature of cuticular resistance to water loss in the adult maize leaf.

Developmental analysis of the adult maize leaf cuticle revealed unexpectedly dynamic changes in cuticle composition from early stages, where cells are dividing, to late stages, where leaf tissue is photosynthetically mature and is exposed to air and light. Integration of results from our biochemical, ultrastructural and functional analyses suggest important roles for wax esters and an ultrastructurally defined, osmiophilic (likely cutin-rich) layer in protection of leaves from dehydration. These studies provide a foundation for future work focused on analysis of underlying gene expression profiles, as well as comparisons between genotypes, aimed at better understanding of the relationship between cuticle composition and function in adult maize leaves and how functionally significant features of the cuticle are supported by underlying gene expression.

## SUPPLEMENTARY DATA

Supplementary data are available online at https://academic.oup.com/aob and consist of the following. Method S1: alkene double bond position analysis. Method S2: determination of alkyl ester calibration response factors. Method S3: enzymatic isolation of abaxial and adaxial cuticles. Figure S1: optimization of wax extraction method for mature maize leaf cuticles. Figure S2: calibration curves for wax ester standards. Figure S3: identification of monoalkenes in maize leaf wax mixtures. Figure S4: changes in the alicyclic compound composition in the developmental gradient of mature maize leaves. Figure S5: comparison of polyester monomers released by transesterification of isolated epidermis and whole leaf tissues. Figure S6: comparison of cuticles of different cell types in the adult maize leaf epidermis at maturity. Figure S7: comparison of adaxial and abaxial surfaces of the adult maize leaf. Table S1: cuticular wax composition of developing maize leaf 8. Table S2: relative isomeric composition of saturated alkyl esters and MS data. Table S3: relative cutin monomer composition along the developing maize leaf 8. Supplementary references.

mcz143_suppl_Supplementary_Table_01

mcz143_suppl_Supplementary_Table_02

mcz143_suppl_Supplementary_Table_03

mcz143_suppl_Supplementary_Material

## FUNDING

This work was supported by NSF Grant IOS-1444507 and by funding from the Canada Research Chairs Program, the Canada Foundation for Innovation (CFI-LOF-31502) and the Natural Sciences and Engineering Research Council (NSERC) of Canada to I.M. TEM work was conducted at the Cellular and Molecular Medicine Electron Microscopy Core Facility at UCSD, which is supported in part by National Institutes of Health Award number S10OD023527.
